# Lipid kinases PIP5Ks and PIP4Ks: potential drug targets for breast cancer

**DOI:** 10.3389/fonc.2023.1323897

**Published:** 2023-12-13

**Authors:** Yue Jin, Jian Xue

**Affiliations:** ^1^ Department of Molecular Diagnosis, Northern Jiangsu People’s Hospital, Yangzhou University Clinical Medical College, Yangzhou, China; ^2^ Department of Emergency Medicine, Yizheng People’s Hospital, Yangzhou University Clinical Medical College, Yangzhou, China

**Keywords:** breast cancer, phosphoinositide, PI(4,5)P2, phosphatidylinositol phosphate kinase, PIP5K, PIP4K

## Abstract

Phosphoinositides, a small group of lipids found in all cellular membranes, have recently garnered heightened attention due to their crucial roles in diverse biological processes and different diseases. Among these, phosphatidylinositol 4,5-bisphosphate (PI(4,5)P2), the most abundant bis-phosphorylated phosphoinositide within the signaling system, stands notably connected to breast cancer. Not only does it serve as a key activator of the frequently altered phosphatidylinositol 3-kinase (PI3K) pathway in breast cancer, but also its conversion to phosphatidylinositol-3,4,5-triphosphate (PI(3,4,5)P3) is an important direction for breast cancer research. The generation and degradation of phosphoinositides intricately involve phosphoinositide kinases. PI(4,5)P2 generation emanates from the phosphorylation of PI4P or PI5P by two lipid kinase families: Type I phosphatidylinositol-4-phosphate 5-kinases (PIP5Ks) and Type II phosphatidylinositol-5-phosphate 4-kinases (PIP4Ks). In this comprehensive review, we focus on these two lipid kinases and delineate their compositions and respective cellular localization. Moreover, we shed light on the expression patterns and functions of distinct isoforms of these kinases in breast cancer. For a deeper understanding of their functional dynamics, we expound upon various mechanisms governing the regulation of PIP5Ks and PIP4Ks activities. A summary of effective and specific small molecule inhibitors designed for PIP5Ks or PIP4Ks are also provided. These growing evidences support PIP5Ks and PIP4Ks as promising drug targets for breast cancer.

## 1 Introduction

Breast cancer, a disease of heterogeneity in the clinic, accounts for approximately 30% of all cancers and remains one of the most significant malignancies in women (1). Prior research has categorized breast cancers into four principal subtypes based on gene expression profiles and the expression of molecular biological markers, including luminal A (positive for estrogen or progesterone receptor ER^+^/PR^+^ with high Ki67 expression), luminal B (ER^+^/PR^+^ with low Ki67 or ER^+^/PR^+^/HER2^+^), HER2^+^(positive for human epidermal growth factor receptor 2) and triple negative (absence of estrogen, progesterone and HER2 receptors) (2, 3). Because of its frequent occurrence of tumor relapse and metastasis, comprehending the fundamental molecular mechanisms and identifying effective molecular biomarkers or anti-tumor targets for breast cancer is imperative.

Recent attention has increasingly focused on phosphoinositides, a small group of lipids found in all cellular membranes. They play crucial roles in diverse biological processes, including cell survival, proliferation, protein and membrane trafficking, cytoskeleton organization, as well as different diseases (4). Those lipid messengers are derived by the phosphorylation of the third, fourth, and fifth positions of the inositol headgroup of phosphatidylinositol (PI), resulting in the generation of seven different phosphoinositide species, like phosphatidylinositol-3-phosphate (PI3P), phosphatidylinositol-4-phosphate (PI4P), phosphatidylinositol-5-phosphate (PI5P), phosphatidylinositol 3,4-bisphosphate (PI(3,4)P2), PI(4,5)P2, phosphatidylinositol 3,5-bisphosphate (PI(3,5)P2) and PI(3,4,5)P3 (5). Alterations in their metabolism underlie various human diseases including neurological disorders, channelopathies, diabetes and cancers (4). Notably, the most clinically advanced strategy targeting phosphoinositide signaling is the PI3K pathway in cancer. It is the most frequently altered pathway in breast cancer, with activation rates as high as 70%. It has been reported to mediate the occurrence of breast tumors, and is closely related to the degree of malignancy, metastasis and drug resistance of the breast tumors (6). In this pathway, PI3K phosphorylates PI(4,5)P2 to generate PI(3,4,5)P3 leading to the subsequent activation of protein kinase B (Akt) and its downstream signaling cascades, which lays the foundation for the application of phosphatidylinositol signaling in cancer research. Several molecules related to PI3K, PI(4,5)P2 and PI(3,4,5)P3 have been effective anti-tumor targets for breast cancer.

Crucially intertwined with phosphoinositide metabolism are phosphoinositide kinases. Multiple phosphoinositide kinases are also therapeutic targets in various human diseases, including cancer, viral infection, neurodegenerative diseases, developmental disorders, diabetes and inflammatory diseases (7). Thus, studies of these kinases can provide new insights into the mechanisms and the extent of their involvement in cancer. Currently, phosphoinositide kinases are broadly categorized into three general families: PI3K superfamily (encompassing all classes of PI3Ks), PIP kinase evolutionary family (including PIKfyve, PIP4Kα/β/γ and PIP5Kα/β/γ) and type II PI4Ks (PI4K2A/B) (7). All PI3Ks are considered therapeutic targets in human diseases, with certain PI3K inhibitors having advanced into clinical trials for breast cancer therapy (8). Beyond PI3Ks, PIP5Ks and PIP4Ks are emerging as an attractive target for therapeutics in inflammation and breast cancer, as they generate PI(4,5)P2 at the plasma membrane, critical for activating the PI3K/AKT signaling pathway. This review focuses on PIP4Ks and PIP5Ks within the PIP kinase evolutionary family, portraying them as intriguing potential targets for breast cancer.

## 2 The phosphoinositide signaling system and related kinases

In recent years, phosphoinositides have gained considerable attention due to their profound influence on a multitude of biochemical processes in eukaryotic cells. Each phosphoinositide localizes distinctly within the cell, performing specific functions. PI serves as the foundational component, primarily synthesized in the endoplasmic reticulum, and is subsequently transported to other membranes via vesicular or nonvesicular lipid transport mechanisms, then delivered by transfer proteins to specific membrane compartments for further phosphorylation by numerous lipid kinases (9, 10). PI3P, PI4P and PI5P are derived by the phosphorylation of PI at the 3rd, 4th and 5th positions, respectively. These phosphoinositides exhibit specific localizations and functions within the cell. For instance, PI3P localizes at early endosomes and acts as a ligand for numerous endosomal proteins. In addition to being catalyzed by PI3KII and PI3KIII, it is also generated from PI(3,4)P2 by 4-phosphatases (INPP4A and INPP4B) (11). Furthermore, PI3P is a substrate for generating PI(3,5)P2 by PIKfyve (12), which also produces the main pool of PI5P in the cell. PI4P, the most abundant monophosphorylated inositol phospholipid in mammalian cells, primarily undergoes phosphorylation in the Golgi complex and the plasma membrane. In the Golgi complex, it plays roles in the biogenesis of transport vesicles (13). However, at the plasma membrane, its primary function is as a substrate for further phosphorylation of PI(4,5)P2. Lastly, PI5P, the latest phosphoinositide discovery, is generated through multiple pathways and serves as a substrate for PI(4,5)P2 production, catalyzed by the enzyme PIP4K. However, the amount of PI(4,5)P2 synthesized from PI5P is small.

As an important bis-phosphorylated phosphoinositide in the phosphoinositide signaling system, PI(4,5)P2 exerts vital cellular functions, as elaborated in subsequent sections. It can be further phosphorylated by PI3K to PI(3,4,5)P3, another lipid with a major role in cell survival, proliferation and cell growth. The ensuing generation of PI(3,4,5)P3 activates Akt and its downstream signaling cascades, laying the foundation for phosphoinositide signaling research in cancer. Conversely, PI(3,4,5)P3 can be converted back to PI(4,5)P2 by the enzyme phosphate and tensin homolog deleted from chromosome 10 (PTEN), which is a tumor suppressor gene and thus becomes a common target of inactivation in breast cancer (14). Overall, PI(4,5)P2 and PI(3,4,5)P3 represent two critical phosphoinositides in cancer. While PI(3,4,5)P3 is typically absent in quiescent cells, it accumulates at the cell membrane upon stimulation with extracellular agonists (15).

**Figure 1:**
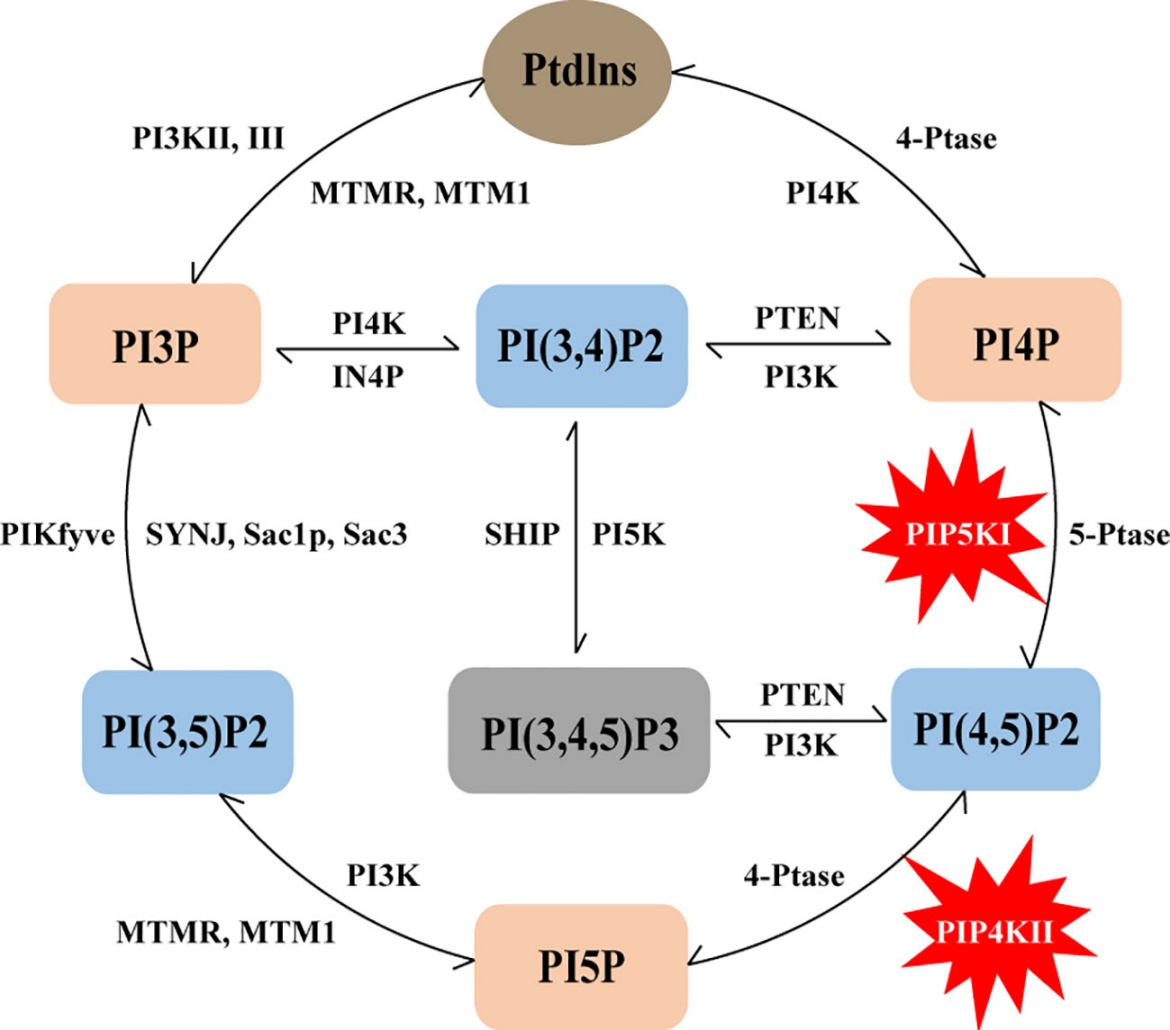
Schematic representation of the phosphoinositide metabolic cycle. PtdIns represents the core structure of phosphoinositides, it can lead to the generation of seven phosphoinositides, including three different mono-phosphorylated PIPs (PI3P, PI4P and PI5P), three bis-phosphorylated PIP2s (PI(3,4)P2, PI(3,5)P2 and PI(4,5)P2) and one tris-phosphorylated PIP3 PI(3,4,5)P3. The lipid kinases, phosphatases and lipases that can produce and convert these phosphoinositides are also indicated in the figure, and PIP5K and PIP4K are specifically labeled.

Numerous studies have significantly advanced our understanding of the roles of these phosphoinositides. It is evident that their levels and functions are intricately regulated by the enzymatic activities of phosphatidylinositol kinases and phosphatases. These enzymes can regulate the synthesis and degradation of multiple phosphoinositides. This intricate interplay is depicted in **Figure 1**. Several kinases have emerged as promising targets for anticancer therapy, and in this context, our focus will be on PI(4,5)P2 and the regulation of its metabolic enzymes, PIP4Ks and PIP5Ks, in the context of breast cancer, as expounded in subsequent sections.

## 3 Insight into the PI(4,5)P2

As mentioned above, PI3K catalyzes PI(4,5)P2 into PI(3,4,5)P3, laying the foundation for the application of phosphatidylinositol signaling in cancer research. PI(4,5)P2 is the central component of the canonical phosphoinositide pathway, and its conversion to PI(3,4,5)P3 is a key event in activating the PI3K/Akt pathway. Conversely, PTEN facilitates the conversion of PI(3,4,5)P3 back to PI(4,5)P2. While PI(3,4,5)P3 is typically scarce in quiescent cells, PI(4,5)P2 abounds, and its relative levels remain relatively constant, barring minor fluctuations during agonist stimulation. The majority of PI(4,5)P2 resides in the plasma membrane (PM), with a substantial pool also observed in the nucleus (16). Within the nucleus, PI(4,5)P2 assumes a non-membranous structure, stabilizing nuclear PI(4,5)P2-binding proteins and participating in nuclear PI(4,5)P2 signaling (17).

PI(4,5)P2, recognized as the most abundant bis-phosphorylated phosphoinositide, serves as the substrate for PI3K, a vital precursor for second messenger inositol-1,4,5-triphosphate (PI(1,4,5)P3), diacylgycerol (DAG) and PI(3,4,5)P3 (7). Extensive studies have underscored the diverse cellular functions orchestrated by PI(4,5)P2, encompassing vesicular trafficking (18), membrane dynamics (15, 19), modulation of ion channel function (20–22), actin cytoskeleton assembly (23, 24), cell polarity (25) and phagocytosis (26). Notably, PI(4,5)P2 influences pre-mRNA splicing machinery and exhibits localization in nuclear speckles, potentially modulating transcriptional processes (27, 28). Furthermore, it has been implicated in cellular migration, a key feature of cancer progression (29, 30). Therefore, it is not surprising PI(4,5)P2 has recently been implicated in breast cancer metastasis. Reduction in plasma membrane PI(4,5)P2 levels, facilitated by specific phospholipases, can enhance cellular migration and metastatic capacity in breast cancer by increasing the abundance of active cytoplasmic cofilin (31). Accumulating evidences also indicate the involvement of PI(4,5)P2 production in the progression of various cancers such as leukemia, melanoma and glioblastoma (32–34).

PI(4,5)P2 generation occurs through the subsequent phosphorylation of mono phosphoinositides from PI, or via the dephosphorylation of PI(3,4,5)P3. It is noteworthy that PI(4,5)P2 can also be produced by the phosphorylation of PI4P or PI5P by two lipid kinase families: PIP5Ks and PIP4Ks. The Type I canonical pathway involves PIP5Ks phosphorylating the 5-position of PI4P to generate PI(4,5)P2, while the noncanonical pathway comprises PIP4Ks phosphorylating the 4-position of PI5P to yield PI(4,5)P2. In mammalian cells, the majority of PI(4,5)P2 is synthesized via the canonical pathway by PIP5Ks, with a smaller pool generated through the noncanonical pathway by PIP4Ks. Although the canonical pathway presumed to be the primary route for PI(4,5)P2 production, PIP4Ks may still generate significant amounts of PI(4,5)P2 at specific cellular locations. Despite generating the same lipid product, these two lipid kinases exhibit diverse biological and metabolic functions, each playing distinct roles, particularly in the context of breast cancer. A detailed discussion of their functions ensues in subsequent sections.

## 4 Insight into the PIP5K and PIP4K family

### 4.1 Location and functions of PIP5Ks and PIP4Ks

PIP5K and PIP4K are emerging as attractive targets for breast cancer due to their ability to produce PI(4,5)P2, which is required for activation of PI3K/AKT signaling pathway. Within this context, PIP5K stands out for its role in generating PI(4,5)P2 by phosphorylation of PI4P at the fifth position of the inositol ring. There are three type I PIP5K isoforms in mammals, including PIP5Kα, PIP5Kβ, and PIP5Kγ (35, 36). These isoforms are encoded by genes *PIP5K1A*, *PIP5K1B* and *PIP5K1C*, respectively. Each isoform exhibits several splice variants, although the specific functions of these variants remain inadequately understood. Remarkably, PIP5K isoforms have distinct cell localizations, translating into unique *in vivo* functions. For example, PIP5Kα is present in membrane ruffles and discrete regions in the nucleus (known as nuclear speckles), regulating the activity of Star-PAP a poly(A) polymerase that controlled the expression of select mRNAs (37). On the other hand, PIP5Kβ primarily resides at the plasma membrane, playing a critical role in receptor endocytosis and cell migration. It is also present on vesicles in the perinuclear region of the cell. PIP5Kγ localizes to focal adhesions and adherens junctions, where it regulates cell polarity and migration of adherent cells and leukocytes (38), Ca^2+^ flux (39), assembly of E-cadherin-based intercellular adhesions, epithelial polarization (40–42), and targets focal adhesions by associating with talin, thereby modulating nascent adhesion formation (43, 44). All three PIP5K isoforms play significant roles in actin remodeling events and various cellular functions, including protein trafficking, cell division and locomotion. Recent evidence has indicated the involvement of PIP5Ks in cancer progression, such as in prostate cancer (45, 46), ovarian cancer (47), and notably, breast cancer (48, 49). Given the critical role of PI(4,5)P2 in regulating cellular events and its contribution to tumor cell invasion and migration, the implication of PIP5Ks in breast cancer is a compelling avenue for investigation.

PIP4K, also comprising three lipid kinase members including PIP4Kα, PIP4Kβ and PIP4Kγ (encoded by *PIP4K2A*, *PIP4K2B* and *PIP4K2C*, respectively) (50, 51), exerts its enzymatic action by phosphorylating PI5P at the fourth position of the inositol ring, resulting in the generation of PI(4,5)P2. Predominantly located in intracellular membranes, the PIP4K isoforms exhibit distinctive cellular distributions and diverse functional roles. Specifically, PIP4Kα is involved in autophagosomes, lysosomes, and peroxisomes, PIP4Kβ primarily resides in the nucleus but can manifest in autophagosomes (52). PIP4Kγ is predominantly found in autophagosomes, Golgi apparatus, and membrane chambers (53). At a sequence level, the isoforms PIP4Kα and PIP4Kβ display a higher homology (83% protein homology), in contrast to PIP4Kγ, which shares approximately 60% homology (50). Due to their sequence differences, the PIP4K isoforms possess varying kinase activities, leading to diverse roles in insulin signaling, receptor recycling, gene regulation, cell stress response and cancers (54–56). PIP4Kα plays a critical role in intracellular cholesterol transport by modulating PI(4,5)P2 homeostasis on peroxisome membranes (57), and it is associated with mTORC2 regulation (58). PIP4Kβ is linked to determining insulin sensitivity and adiposity (59). Both PIP4Kα and PIP4Kβ are shown to be required for autophagosome-lysosome fusion during metabolic stress (60). Differently, PIP4Kγ positively regulates Notch1 signaling by facilitating receptor recycling (61). It contributes to increased inflammation characterized by decreased regulatory effectors cells (62), and plays crucial roles in T-cell signaling (63). Recent findings have established a link between PIP4Kγ and mTORC1, demonstrating their interaction in a self-regulated feedback loop to maintain low and tightly regulated mTORC1 activation during starvation (64). Besides the above functions that have already been discussed, these kinases have been implicated in several diseases, including acute leukemias, glioblastoma, soft tissue sarcomas, among others. Notably, there is a burgeoning interest in targeting PIP4Ks for therapeutic interventions in cancer (65). Among them, the first study to implicate PIP4Ks in cancer and for setting the stage for oncological research is in breast cancer (66).

### 4.2 The expression of PIP5Ks and PIP4Ks in breast cancer

**Figure 2:**
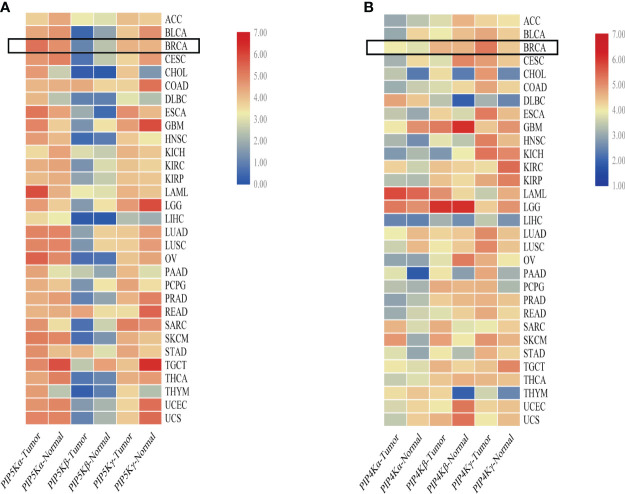
Comparative overview of PIP5K and PIP4K isoforms expression in multiple cancers. **(A)** shows the PIP5Ks expression in multiple cancers. **(B)** represents the expression of PIP4Ks. The heat map is organized based on the difference in median expression between the tumor samples and the paired normal tissue samples. The expressions of PIP5Ks and PIP4Ks in breast cancer are highlighted with a black box. ACC, adrenocortical carcinoma; BLCA, bladder urothelial carcinoma; BRCA, breast invasive carcinoma; CESC, cervical and endocervical cancers; CHOL, cholangiocarcinoma; COAD, colon adenocarcinoma; DLBC, lymphoid neoplasm diffuse large B-cell lymphoma; ESCA, esophageal carcinoma; GBM, glioblastoma multiforme; HNSC, head and neck squamous cell carcinoma; KICH, kidney chromophobe; KIRC, kidney renal clear cell carcinoma; KIRP, kidney renal papillary cell carcinoma; LAML, acute myeloid leukemia; LGG, brain lower grade glioma; LIHC, liver hepatocellular carcinoma; LUAD, lung adenocarcinoma; LUSC, lung squamous cell carcinoma; OV, ovarian serous cystadenocarcinoma; PAAD, pancreatic adenocarcinoma; PCPG, pheochromocytoma and paraganglioma; PRAD, prostate adenocarcinoma; READ, rectum adenocarcinoma; SARC, sarcoma; SKCM, skin cutaneous melanoma; STAD, stomach adenocarcinoma; TGCT, testicular germ cell tumors; THCA, thyroid carcinoma; THYM, thymoma; UCEC, uterine corpus endometrial carcinoma; UCS, uterine carcinosarcoma.

To comprehensively understand the expression of PIP5K and PIP4K in multiple cancer (breast cancer included), we conducted an analysis comparing median expression levels between tumor samples and their paired normal tissue counterparts using the Gene Expression Profiling Interactive Analysis (GEPIA) dataset. As depicted in **Figure 2A**, PIP5K exhibits ubiquitous expression, with PIP5Kα demonstrating higher expression levels than the other two isoforms in breast cancer. PIP5Kα, a key upstream factor of PI3K/AKT pathway, is notably expressed at elevated levels in triple-negative breast cancers and associated with poor patient outcome (67). Its expression is imperative for invadopodia formation and promotes survival of breast cancer cells (68). Studies have also reported that the PIP5Kα-dependent pool of PI(4,5)P2 promotes breast cancer cell proliferation (49). Overexpression of *PIP5K1B* in MCF-7 breast cancer cells results in cell scattering and the stimulation of cell migration and invasion (69). Moreover, a significant correlation between PIP5Kγ expression and the progression of breast cancer is also observed (70). Notably, it is overexpressed in triple-negative breast cancer (71). Depletion of PIP5Kγ inhibits cell proliferation, MMP9 secretion, cell migration and invasion of breast cancer cells (48). Additionally, the expression of its 90 kD splice variant (PIP5Kγ90) also enhances the migration, invasion and proliferation of breast cancer cells (72).

When studying the PIP4Ks, both PIP4Kα and PIP4Kβ exhibit elevated expression levels in breast cancer compared to normal tissues (66). Their downregulation significantly suppresses the proliferative capacity of triple negative breast cancer cell lines and dramatically diminishes the cellular viability. Intriguingly, deletion of these two isoforms in mice suppresses tumor formation in the presence of Trp53 deletion (66). For *PIP4K2B*, both excessively high and low levels are implicated in breast cancer and patient survival. Overexpression of *PIP4K2B* can increase breast cancer cell proliferation and anchorage-independent growth in different subsets of breast cancer cell lines (73). Additionally, a correlation between low *PIP4K2B* expression in human breast tumors and reduced patient survival is found (74). *PIP4K2B* is also reported to be co-amplified with the proto-oncogene ERBB2 gene. Notably, a striking co-occurrence between high *PIP4K2A* expression as well as *PIP4K2B* amplification with TP53 mutation/deletion is even showed. In contrast to PIP4Kα and PIP4Kβ, PIP4Kγ, while approximately 1% as active as the former isoforms, displays higher expression in breast cancer based on a comparative overview of PIP4K isoform expression in multiple cancers (**Figure 2B**). This underscores PIP4Kγ as a potential drug target for breast cancer treatment.

### 4.3 Regulation of PIP5Ks and PIP4Ks

Many studies have demonstrated various mechanisms of the regulation of PIP5K activity, including spatiotemporal regulation and other protein stability. When it comes to spatiotemporal regulation, PIP5K is intricately modulated by a cadre of upstream regulators, including Rho family of GTPases, binding partner proteins and post-translational modifications such as phosphorylation (75). For instance, elevated level of PIP5Kα increases expression of pSer-473 AKT and is in complexes with VEGFR2, acting as co-factor of ER-alpha to regulate activities of target genes, including cyclin D1 and CDK1, particularly in triple-negative and ER^+^ breast cancer cells (67). As a key player downstream of EGFR, a tight association between EGFR and PIP5Kγ in the progression of breast cancer is found. Phosphorylation modifications of PIP5Kγ by EGFR profoundly impacts tumor formation and metastasis. Cdk5-mediated PIPKIγ90 phosphorylation exerts spatiotemporal control over PI(4,5)P2 production and fibronectin secretion, intricately regulating cancer cell invasion (76). The interplay of PIP5Kγ and PI3K, orchestrated by Src-mediated regulation, spatially generates PI(4,5)P2 and PI(3,4,5)P3, culminating in subsequent Akt activation (71). Contrasting this perspective, a study proposed an alternative regulatory mechanism involving the neural precursor cell expressed, developmentally down-regulated gene 4 (NEDD4)-dependent control of PIP5K protein stability, affecting PIP5K-dependent PI(4,5)P2 generation. Specifically, they suggest that PIP5Kα increases the plasma membrane PI(4,5)P2 level and contributes to breast cancer cell proliferation through PI3K/Akt activation, while the whole process can be negatively controlled by the ubiquitin ligase NEDD4, which can interact with the C-terminal region and ubiquitinate PIP5Kα (49). In addition, PIP5Kγ is implicated in the transcriptional upregulation of the PD-L1 gene by activating the NF-κB pathway in the triple negative breast cancer (77).

Concerning PIP4Ks, they are often negatively regulated by phosphorylation. Protein kinase D (PKD)-mediated phosphorylation of PIP4Kα at Thr376 and its mutation to aspartate significantly reduces the enzymatic activity of PIP4Kα (78). Additionally, the protein kinase formerly casein kinase 2 (CK2) can phosphorylate the Ser304 site of PIP4Kα, intriguingly, mimicking phosphorylation by mutating the residue to aspartate induces a translocation from the cytoplasm to the plasma membrane instead of a change in the PIP kinase activity (79). PIP4Kβ, on the other hand, can be phosphorylated by the p38 stress-activated protein kinase at Ser326 directly, resulting in inhibition of PIP4Kβ activity (80). Furthermore, mTORC1 can phosphorylate PIP4Kγ at Ser324 and Ser328. In addition to the phosphorylation mentioned above, the activity of PIP4Ks is intricately modulated by interactions with other proteins, such as the proline isomerase Pin1 (81). Moreover, the regulation of PIP4Kβ is involved in ubiquitylation, where it acts as a key regulator of the p38 MAPK pathway, interacting with the ubiquitin ligase complex Cul3-SPOP (SPOP) and co-localizing at nuclear speckles (82).

### 4.4 Inhibitors of PIP5Ks and PIP4Ks

PIP5Ks has been hypothesized to provide a potential therapeutic target of interest in the treatment of cancers. To comprehensively comprehend and exploit the role of PIP5Ks in human cancers, some potent and specific PIP5K inhibitors have been reported, with some currently in early stages of preclinical development. Notably, ISA-2011B, a diketopiperazine fused C-1 indol-3-yl substituted tetra-hydro-isoquinoline, stands out as a well-described PIP5K inhibitor, specifically inhibiting the PIP5Kα-associated AKT pathways (45). However, further optimization is required due to its significant off-target effects. 5-(cyclohexanecarboxamido)-2-(phenylamino)thiazole-4-carboxamide, termed as UNC3230, has been identified as an inhibitor targeting PIP5Kγ (83). At the same time, it also targets PIP4K and shows higher potency towards PIP4Kγ over PIP5Kγ in *in vitro* lipid kinase assays. Moreover, several high-quality *in vitro* tool compounds have been identified (84). Despite the existence of some inhibitors, searching for more highly effective PIP5K inhibitors as tool compounds is still valuable.

Given the current roles of the PIP4Ks in cancers, they have also emerged as attractive drug targets in the context of cancers, with their inhibitors being actively explored in preclinical studies. At present, various PIP4K inhibitors, encompassing pan inhibitors and isoform specific inhibitors, have been reported. Compound CC260, a highly potent and selective noncovalent dual inhibitor for both PIP4Kα and PIP4Kβ, exhibits promising potential by disrupting cell energy homeostasis and activating AMPK while inhibiting mTORC1, selectively targeting p53 mutant breast cancer cells (85). However, it is important to note its off-target activity against PI3K-δ. THZ-P1-2, a pan-PIP4K inhibitor, targets cysteines in a disordered loop of isoforms PIP4Kα/β/γ, and it is shown to have anti-proliferative activity (86). In addition, PIP4K lipid kinases have been identified as the target of A131 and CVM-05-002 (87, 88). The former selectively regulates the cell cycle entry of Ras-activated cancer cells and induces reversible growth arrest in normal cells by transcriptionally upregulating *PIK3IP1*. Except for the inhibitors mentioned above, several isoform-specific PIP4K inhibitors have also been identified. For example, I-Ome Tyrphostin AG-538 is identified as the ATP-competitive inhibitor of PIP4Kα (89). BAY-091 and BAY-297 serve as the valuable inhibitors of the kinase PIP4K2A (90), ARUK2002821 is also identified as an selective PIP4Kα inhibitor and has broad selectivity against lipid and protein kinases (91). Another noteworthy inhibitor is SAR088, representing the first orally available and *in vivo* active PIP4K2B inhibitor (92). The quinazolin-4-amine compound NIH-12848 and its derivative compound 40 are putative PIP4Kγ inhibitors, and they are likely to interact with the PI5P-binding site of PIP4Kγ (93, 94). NCT-504 have also been disclosed (95). These diverse inhibitors collectively present a rich landscape for potential therapeutic interventions targeting PIP4Ks in cancer. These effective and specific small molecule inhibitors designed for PIP5Ks or PIP4Ks are summarized in **Table 1**.

**Table 1:** Summary of preclinical inhibitors of the PIP5Ks and PIP4Ks.

Compound	Target	Description
ISA-2011B	PIP5Kα	•It significantly inhibits growth of tumor cells by targeting PIP5Kα-associated PI3K/AKT and the downstream survival, proliferation, and invasion pathways. •It has significant off-target effects on the class I PI3K p110α.
UNC3230	PIP5Kγ PIP4Kγ	•It is competitive against ATP with an average Ki of 23 nM, and shows higher potency towards PIP4Kγ over PIP5Kγ *in vitro* lipid kinase assays. •It can hit other lipid and/or protein kinases, also has a narrow efficacy window and low solubility in appropriate vehicles.
Series of 4-aminopyridine derivatives (compounds 8, 20, 25)	pan-isoform PIP5K	•It shows the considerable optimization of both potency for PIP5K, and selectivity over the closely related kinase PI3Kα. •The compounds have the anticipated effects in downstream cellular signalling assays as well as demonstrating a highly specific effect on modulating PI(4,5)P2 cellular levels.
I-OMe Tyrphostin AG-538	PIP4Kα	•It is an ATP-competitive inhibitor of PIP4Kα with an IC50 of 1µM.
ARUK2002821(36)	PIP4Kα	•It is a potent inhibitor of PIP4Kα, selective to PIP4Kβ and PIP4Kγ, and acts as a target for use in cells at submicromolar concentrations.
BAY-091 and BAY-297	PIP4Kα	•They are potent and selective chemical probes, also the 1,7-naphthyridinebased inhibitors.
NIH-12848 and its derivative compound 40	PIP4Kγ	•It is an allosteric non-ATP-competitive inhibitor that binds to the putative PI5P substrate binding site. •It inhibits the translocation of Na^+^/K^+^-ATPase to the plasma membrane and prevents reversibly their forming of ‘domes’.
SAR088	PIP4Kβ	•It is a relatively specific inhibitor and exhibits reasonable physico-chemical properties, no liver CYP34A inhibition, as well as intermediate cell-permeability and high metabolic stability. •It demonstrates exposure in plasma, muscle and liver tissues. •It lowers blood glucose levels.
THZ-P1-2 and its derivative compound 30	pan-PIP4K	•It targets conserved cysteines outside the ATP-binding pocket of the PIP4K kinase domain. •Its treatment reduces proliferation and impaired autophagy in cancer cells.
A131	pan-PIP4K	•It inhibits PIP4Ks and causes reversible growth arrest in normal cells by transcriptionally upregulating *PIK3IP1.* •It has poor aqueous solubility and a relatively short half-life after intravenous administration, limiting its *in vivo* use.
CVM-05-002 derivative compound 13	pan-PIP4K	•It is a potent pan-PIP4K inhibitor with excellent kinome-wide selectivity, replacing the rhodanine-like moiety present in CVM-05-002 with an indole.
CC260	PIP4Kα/β	•A potent and selective small-molecule probe disrupts cell energy homeostasis, causing AMPK activation and mTORC1 inhibition. •It has several off-target activities for other protein and lipid kinases, like PI3K-δ.

## 5 Conclusion and perspective

In recent years, there has been a surge in research on phosphoinositide signaling, acknowledging its critical role in fundamental cellular processes and its close relationship with various diseases. However, for a comprehensive understanding and effective utilization of phosphoinositides, further cellular and physiological studies are imperative to unravel the intricacies of phosphoinositide signaling pathway and their associated enzymes. From the current perspective, lipid kinases PIP4Ks and PIP5Ks are emerging as promising therapeutic targets in breast cancer, but, clearly, a thorough exploration of their roles and mechanisms across diverse breast cancer subtypes necessitates extensive experimental investigation. Currently, distinct isoforms within the PIP5K/PIP4K family exhibit varying distribution and expression patterns in cells and tissues, implying their potentially divergent roles in different stages and subtypes of breast cancer. This underscores the importance of analyzing and approaching treatment strategies for triple-negative, ER^+^ or other breast cancer types differently, taking into account the unique characteristics of these isoforms. Among these isoforms, particularly, PIP4Kγ, despite its perceived lower activity, exhibits higher expression than the other PIP4K isoforms in breast cancer, warranting focused attention in future studies due to the limited research on this isoform in the context of breast cancer.

Further functional comprehension of PIP5Ks and PIP4Ks in breast cancer necessitates a deeper investigation into the regulation. While spatiotemporal regulation is a relatively well-studied mode of PIP4K/PIP5K regulation, in fact, attention should broaden to encompass the influence of other proteins and environmental factors on these kinases. At the same time, understanding their substrate PI5P and product PI(4,5)P2 is crucial, given their significance in the PI3K pathway. Especially the PI(4,5)P2, it is not only the key to activate the PI3K pathway, but also its conversion to PI(3,4,5)P3 hold substantial promise for breast cancer research. In the following studies, we can focus on the regulatory process between PIP5Ks/PIP4Ks and PI(4,5)P2.

Moreover, a key area of focus is the development of inhibitors targeting PIP5Ks and PIP4Ks. These inhibitors enable validation of the functions of these kinases and present promising avenues for future therapeutic interventions in breast cancer. Despite the existence of effective and specific small molecule inhibitors, enhancing specificity and overall pharmacokinetics while mitigating off-target activity is paramount. Considering targeting multiple PIPK families and isoforms, and designing inhibitors based on their unique properties, can address issues of off-target activity and drug resistance, thus enhancing the prospects of therapeutic efficacy. For example, PIP4Ks can bind to both ATP and GTP, but PIP4Kβ preferentially utilizes GTP rather than ATP for PI5P phosphorylation, which allows us to design ATP or GTP binding sites accordingly. In addition, the current common strategy for developing kinase inhibitors is to inhibit substrate phosphorylation by occupying the ATP-binding site of the drug molecule. However, ATP binding sites are highly conserved in kinases, which makes the development of competitive ATP inhibitors with high selectivity a huge challenge. Considering the highly differentiated substrates and the uniqueness of their binding sites in terms of geometric and electrostatic properties, targeting unique substrate binding sites and blocking substrate binding appears to be a superior strategy for inhibiting PIPK families. Meanwhile, we should also consider additive or synergistic effects between inhibitors with different working mechanisms.

In conclusion, the expression pattern and function of PIP5K and PIP4K in breast cancer underscore these two kinases as attractive targets for therapeutics in breast cancer. However, our current understanding of PIP5K and PIP4K is still limited, necessitating future studies aimed at unraveling the mechanisms specific to each isoform. This deeper understanding will pave the way for effective clinical applications of these kinases in breast cancer treatment.

## Author contributions

JX: Writing – original draft. YJ: Writing – review & editing.
